# Systematic review of economic evaluations of interventions for high risk young people

**DOI:** 10.1186/s12913-018-3450-x

**Published:** 2018-08-23

**Authors:** Kim Edmunds, Rod Ling, Anthony Shakeshaft, Chris Doran, Andrew Searles

**Affiliations:** 1 Hunter Medical Research Institute, University of Newcastle, Lot 1, Kookaburra Circuit, New Lambton Heights, NSW 2305 Australia; 2 National Drug and Alcohol Research Centre, University of New South Wales, Randwick Campus, 22-32 King Street, Randwick, NSW 2031 Australia; 3 Centre for Indigenous Health Equity Research, Central Queensland University, 160 Ann Street, Brisbane, QLD 4000 Australia

**Keywords:** High risk young people, Multi-component intervention, Economic evaluation, Cost-benefit analysis, Cost-effectiveness analysis, Cost-utility analysis, Social return on investment

## Abstract

**Background:**

The aim of this systematic literature review is to identify and critique full economic evaluations of interventions for high risk young people with the purpose of informing the design of future rigorous economic evaluations of such intervention programs.

**Methods:**

A PRISMA compliant search of the literature between 2000 and April 2018 was conducted to identify full economic evaluations of youth focussed interventions for at risk young people. Duplicates were removed and two researchers independently screened the article titles and abstracts according to PICOS criteria for exclusion and inclusion. The remaining full text articles were assessed for eligibility and a quality assessment of the included articles was conducted using the Drummond checklist.

**Results:**

The database, grey literature and hand searches located 488 studies of interventions for at risk young people. After preliminary screening of titles and abstracts, 104 studies remained for full text examination and 29 empirical studies containing 32 separate economic evaluations were judged eligible for inclusion in the review. These comprised 13 cost-benefit analyses (41%), 17 cost-effectiveness analyses (53%), one cost-utility analysis (3%) and a social return on investment (3%). Three main methodological challenges were identified: 1. attribution of effects; 2. measuring and valuing outcomes; and 3. identifying relevant costs and consequences.

**Conclusions:**

A cost-benefit analysis would best capture the dynamic nature of a multi-component intervention for high risk young people, incorporating broader intersectoral outcomes and enabling measurement of more domains of risk. Prospective long-term data collection and a strong study design that incorporates a control group contribute to the quality of economic evaluation. Extrapolation of impact into the future is important for this population, in order to account for the time lag in effect of many impacts and benefits arising from youth interventions.

**Electronic supplementary material:**

The online version of this article (10.1186/s12913-018-3450-x) contains supplementary material, which is available to authorized users.

## Background

The transition from childhood to adulthood is typically marked by important milestones such as high school graduation or entry into the labour force that contribute to identity formation, self-assurance and capacity building. Most young people experience relatively few harms during this transition and for young people who do experience harms, the majority are the result of temporary risk factors, such as experimenting with substance use or delinquent behaviours [1]. However, a relatively small proportion of young people experience multiple and sustained risk factors which manifests in multiple harms such as poor mental health, cognitive detriments [2, 3], substance abuse, violence, risky sexual behaviour, unintentional injury, low engagement with education and employment, poor dietary practices, crime and incarceration [4–6]. The occurrence of multiple risk factors is typically associated with social determinants of poor health, such as low socioeconomic status, family dysfunction, lack of appropriate housing or homelessness, racism, a lack of cultural identity, systemic discrimination and social exclusion [1, 3, 4, 7].

Bonds with society for these young people are already attenuated and adolescence further compounds this effect, creating heightened potential for antisocial behaviours and a considerable negative impact on their lifetime trajectory [8]. For example, young people who have experienced abuse or neglect are more likely to engage in heavy drinking and illegal drug use, which is associated with antisocial behaviour and crime [3, 6, 9]. They tend to have poor lifestyle practices, engage in risky sexual behaviour and violence and, therefore, have poor physical and mental health. They are also more likely to have cognitive detriments and low levels of educational achievement and engagement, all of which contribute to reduced workforce participation and reduced lifetime earnings [6, 10].

This complex set of interrelated and mutually reinforcing factors imposes considerable social and economic costs on the individual, community and society [4, 10, 11]. Although the benefits of effective and efficient interventions for reducing harms associated with long term antisocial behaviours among high risk young people have long been recognised in principle [6, 12], most interventions that have been evaluated have either addressed only a limited number of risk factors or have not been shown to be effective [1, 4, 13]. A current systematic review of studies examining interventions for high risk young people has identified that only 5% of interventions targeted multiple risk factors, and none incorporated an economic evaluation [4]. This lack of evidence is surprising given the considerable potential for substantial personal, social and economic benefit from even modest reductions in risk, particularly since the clustering of multiple risk factors means that a reduction in one outcome may spill over into others [14]. These benefits derive from increased productivity and contribution to community, as well as reduced impacts on family, health costs, crime and justice system costs, and welfare dependency [15].

Effective social policy assists high risk young people to access economic opportunities, so they can enjoy improved quality of life and are less likely to impose an economic, health and social burden on society. However, such policies require the availability of adequate resources to provide assistance, and interventions that cost effectively use these resources to reduce harms. The objective of economic evaluation is to identify, measure and value what society forgoes when it funds an intervention (the opportunity cost) and what it gains (the benefit) [16]. Economic evaluation thus provides an important evidence base for decision making in the health-care sector, aiding policy makers in the allocation of society’s scarce resources [16]. The dearth of evidence around the effectiveness and economic efficiency of social interventions for high risk young people provided the impetus for this systematic review.

### Aim

The aim of this systematic literature review is to identify and critique full economic evaluations of interventions for high risk young people with the purpose of informing the design of future rigorous economic evaluations of such intervention programs.

## Methods

### Identification - search strategy

A PRISMA compliant systematic search of the literature was conducted with the assistance of an accredited librarian [17] (see Additional file 1). The search comprised two steps as per the guidelines provided in the Cochrane Collaboration Handbook on Systematic Reviews of Interventions [18]. First, a preliminary search was conducted to identify original articles in the following electronic databases: Econlit, PubMed Clinical Queries and Scopus. A full search was then conducted in CINAHL, Embase, Global Health, Medline, PsychInfo and Social Work Abstracts. Given that the literature often refers to the need for more economic evaluations in the area of youth programs [14, 19, 20], coupled with the lack of economic evaluations of interventions for high risk young people that address multiple domains in the literature [4], the terms used in the search strings to identify high risk young people and interventions were designed to generate as many relevant interventions as possible. Terms for full economic evaluations were then added to the search strings. Full economic evaluation is used here to refer to cost-benefit analysis (CBA), cost-effectiveness analysis (CEA), cost-utility analysis (CUA) and social return on investment (SROI), a type of CBA [16]. Searches were limited to English language and the years 2000-April 2018.

Second, a grey literature search was conducted to identify articles not located by the electronic database search. Search terms for high risk young people and full economic evaluations were applied to a search of the following websites: Centre for Aboriginal Economic Policy Research (CAEPR), National Institute for Health and Care Excellence (NICE), Health Technology Assessment, Washington State Institute for Public Policy (WSIPP), HealthInfoNet, Closing the Gap Clearinghouse, LIt.search, Australian Policy Online (APO), Virginia Commonwealth University Library, Google and Google Scholar. A hand search of reference lists of identified articles was also conducted.

**Fig. 1:**
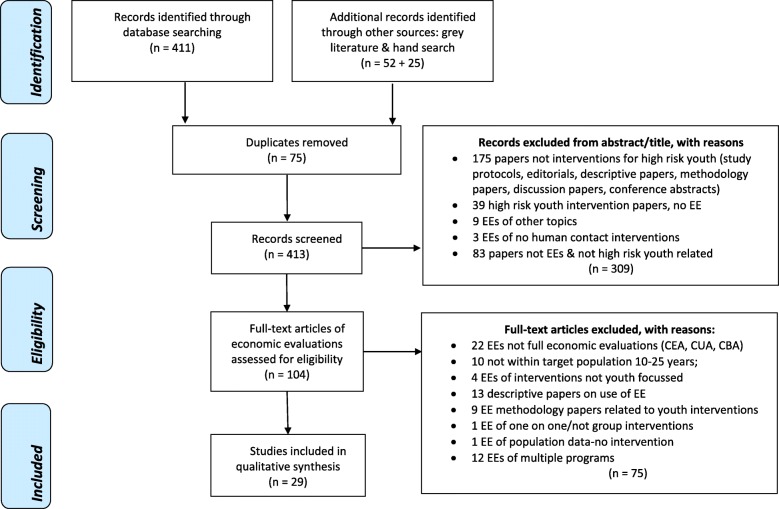
PRISMA flow diagram

Figure 1 shows the search process, from the databases searched to identify records to the exclusion criteria applied in the initial screening, and finally, the eligibility criteria used to assess full text articles for inclusion in the review.

### Step 1: Screening

Two reviewers (KE, RL) independently screened the titles and abstracts of retrieved articles following the PICOS criteria for inclusion & exclusion specified in Table 1.

**Table 1:** PICOS criteria for inclusion and exclusion of studies

Parameter	Inclusion criteria	Exclusion criteria
Population	High risk young people between the ages of 10–25	Not youth intervention
Intervention	Any youth focussed intervention	Not involving contact with youth
Comparator	Treatment as usual	
Outcomes	Any of the five domains of risk (criminal activity, education and employment, homelessness, mental health & wellbeing, sexual behaviour, substance abuse; & violence)	
Study design	Full economic evaluation (CEA, CUA, CBA, SROI)	Not economic evaluation/not youth related

### Step 2: Eligibility criteria

After excluding articles that did not match the inclusion criteria, the full-text versions of the remaining articles were obtained and assessed for eligibility.

#### Key characteristics of identified economic evaluations

Data extracted from the 29 eligible studies was guided by the Cochrane Handbook for Systematic Reviews of Health Interventions [18] and is presented in Additional file 2. For each study, information was thus recorded on: first author, year and country of publication; sample size and setting; type of study or intervention, the outcomes addressed in the economic evaluation, type of economic evaluation and finally, key findings of the study and methodological insights. Information was also extracted on the domains of risk targeted by the intervention (criminal activity; education and employment; mental health & wellbeing; sexual behaviour; substance abuse; and violence) adopted from Knight et al. [4]. In line with the aims of this review, the studies are grouped according to the type of economic evaluation: 1. CBA and SROI, and 2. CEA and CUA. In order to enable comparison between the outcomes of like economic evaluations, within these two larger groups, economic analyses are grouped according to the domain(s) of risk addressed in the study.

#### Appraisal of the methodological quality of the economic evaluations

The quality of the included economic evaluations was rated using the Drummond checklist [16]. The Drummond checklist was designed to guide the critique of economic evaluations and considers: 1) the research question; 2) the description of the study/intervention; 3) the study design; 4) the identification, 5) measurement, and 6) valuation of costs and consequences; 7) whether discounting was carried out; 8) incremental analysis; 9) presentation of results with uncertainty and sensitivity analyses; and 10) discussion of results in the context of policy relevance and existing literature. A rating scale, developed by Doran [21], was utilised to attribute a potential score of 1 to each of the items on the checklist. The aggregate results provide an economic quality appraisal of poor (1–3 points), average (4–7 points) and good (8–10 points). Authors KE and RL conducted independent quality appraisal of the included economic evaluations. The detailed Drummond checklist quality appraisal is presented in Additional file 3.

## Results

The combined searches of all databases located 411 studies. Fifty two additional records were identified from the grey literature search. The hand search generated a further 25 records. After preliminary screening of titles and abstracts, 75 duplicates and 309 studies that did not match the inclusion criteria were removed. A total of 104 studies remained for full text examination and 29 studies were judged eligible for inclusion in the review.

The included economic evaluations comprise 13 CBAs, one SROI, 17 CEAs and one CUA, a total of 32 from 29 publications. Three studies each conducted both a CBA and a CEA. The majority of economic evaluations that met the inclusion criteria were published in the US (*n* = 20), followed by Australia (*n* = 3), the UK (*n* = 3), the Netherlands (*n* = 2), and Germany (*n* = 1). The summary results of included economic valuations are presented in Additional file 2.

### Methodology of economic evaluations

Despite all included economic evaluations being rated average or good (Additional file 3), many suffered from methodological limitations, emphasising the challenges often faced in the conduct of economic evaluations of public health/community interventions. A number of factors impact on the reliability of conclusions drawn in economic evaluations such as uncertainty in effect size, accuracy of cost information, scope, modelling and timing of the analysis, perspective and choice of discount rate. A considerable number of included studies failed to follow best practice for conduct of economic evaluations and did not adjust for differential timing, or perform incremental or uncertainty analysis. However, three key methodological challenges were identified from the literature as representative of the methodological issues more specific to the conduct of economic evaluations of interventions for youth populations: 1). attribution of effects; 2). measuring and valuing outcomes; and 3). identifying relevant costs and consequences.

Attribution of effects (1.) refers to the strength of the evidence around causality and is reflected in the range of study designs. RCTs are the preferred source of evidence to determine estimates of intervention effects, but these are often difficult to conduct in a public health environment (as opposed to more controlled clinical or laboratory settings), so alternative approaches are sometimes needed. In addition, outcomes are often measured in the short term whereas the impact of a public health intervention may occur in the long term. Measuring and valuing outcomes (2.) refers to the approach taken in an economic analysis. In health, for example, the outcome of interest may be quite narrow and directed at specific individuals or groups such as cost per life years gained for a treatment or cost per cases prevented for immunisation and require a CEA. Alternatively, many public health interventions have effects on individuals outside the target group and thus  need a broader measurement of outcomes, requiring a CBA. Finally, identifying relevant costs and consequences (3.) refers to which costs and benefits are included in an economic evaluation. Some studies may not have collected the data required by an economic evaluation thus limiting the cost and consequences that can be identified, or necessitate modelling based on assumptions derived from other sources or the literature. The impact of public health interventions is often broad and can have a ripple effect, so the costs and benefits may be related to a number of sectors and this needs to be addressed in the economic evaluation. It is these three methodological challenges that will form the focus of this review.

The methodological characteristics of the included economic evaluations are described below. Firstly, the methodology of the CBAs and SROI will be examined, followed by the CEAs and CUA, in order to best facilitate comparison across like studies.

### Cost-benefit analyses and social return on investment

The review identified 13 CBAs and one SROI of interventions for at risk young people [22–34].

#### Attribution of effects

Of these economic analyses, ten were retrospective [22–24, 27, 29–34], two were prospective [25, 35], one hypothetical [26, 36] and one preliminary (i.e. based on initial, short term data collection) [28]. The intervention study designs varied from RCTs [23, 30, 32–34], clinical trials (not RCT) [27], quasi-experimental designs [25, 28, 31, 35] to designs with no control group [22, 24, 26, 29]. For many of the studies on which the included economic analyses were based, outcome data was collected over a short term [22, 23, 27–29, 31, 34, 35]. Some studies used modelling or extrapolation of data over a longer duration to demonstrate benefit [24, 28, 31, 33, 34]. Five studies only had access to longer term outcome data ranging from 5 to 13.7 years post intervention [24, 25, 30, 32, 33].

The analyses varied considerably in approach. For example, a CBA based on a RCT with long term follow up data (13.7 yrs), taxpayer and victim perspectives and a comprehensive collection of costs [32] compared to a SROI of the Ganbina program employing a societal perspective which included all stakeholders, based on only 12 month data, no control group and paucity of data in some areas [22].

#### Measurement and valuation of outcomes

The number of outcomes measured and valued varied widely from one to 19. Ten of these analyses measured and valued three or less outcomes [23, 24, 26–28, 31–35]. Outcomes varied widely and included reduced substance use, increased educational attainment, reduced criminal activity, teenage births averted, improved productivity/employment, increased social networks, improved mental and physical health, reduced acts of delinquency, increased days of abstinence, reduced absenteeism, reduced arrests, averted STIs, and increased condom use. Many analyses were confronted with a lack of data, so measurement or valuation of outcomes often used the work of previous studies. For example, Belfield [23] used existing costs of crime [36] to derive monetary values for being a heavy drug user, a high school drop-out or a career in juvenile crime. Hoeflmayr and Hanewinkel [31] used prevalence measures from their effectiveness study applied to a combination of two existing progression models [37, 38] to determine prevention of lifetime established smokers.

#### Costs and consequences

In general, a broader perspective, which usually incorporates a number of costs and consequences, is associated with a CBA, and in the 13 included CBAs and the SROI, nine used the broadest: a societal perspective [22–26, 29, 31, 33, 34]. Of these nine, however, two included narrower perspectives such as youth participants, mentors, taxpayer or government [23, 26]. The remaining studies used narrower perspectives such as taxpayer and victim perspectives [27, 32], an employer perspective [30], a local justice system perspective [35] and the remaining analysis did not state the perspective, but a health provider perspective is inferred [28]. While some analyses were conducted from a societal perspective and identified a broad range of costs and consequences, they restricted the costs (e.g. direct costs only) [25] and benefits included in the analysis (e.g. delinquency & tobacco use only) [33].

### Cost-effectiveness analyses and cost-utility analysis

The review identified 17 CEAs and one CUA of interventions for at risk youth.

#### Attribution of effects

Of these 18 included economic analyses, 15 were retrospective analyses [30, 34, 37, 39–50], one was prospective [51] and two hypothetical [26, 52]. Intervention study designs were predominantly RCTs (*n* = 9) [30, 34, 37, 39, 44, 48, 50, 51], four were modelled using various sources of data (clinical trials, government surveys, literature) [26, 42, 43, 52], four were quasi-experimental designs [40, 41, 47, 49] and one had no control group [46]. Not unlike the CBAs examined above, the majority of the CEAs and the CUA had access only to short term data. In those studies where data was collected, final follow up took place at 7 months [34, 43], 12 months [39, 41, 45, 47, 48], 18 months [50], 2 years [37, 40, 49, 51] and 5–6 years [30, 46]. CEAs varied in approach and their ability to attribute effect to the intervention of interest. For example, two studies of smoking prevention programs employed very different analyses. A prospective CEA based on a cluster RCT had a large sample size (*n* = 10730), two year follow up data, was based on an ITT analysis and collected costs on all resources relevant to the public sector perspective used [51]. A retrospective CEA of the Full Court Press (FCP) project, which also had a large sample size (n= 7725), had no control group, five year follow up data, real life costs and used a public health perspective [46]. This analysis used youth specific smoking participation price elasticity and the ratio of the FCP project costs and the costs of other tobacco control programs used to come up with a 68% attribution of effect to FCP.

#### Measurement and valuation of outcomes

As CEAs tend to have narrower application and are relevant to interventions that have a common effect of interest, it is unsurprising that the CEAs in this review generated a variety of outcome measures such as number of incidences of substance abuse [48]; initiation of tobacco use, instances of delinquent behaviour [33]; decreases in burden of disease for mental health [26]; number of (established) smokers prevented [31, 37]; days of abstinence, per cent of adolescents in recovery [39]; reductions in tobacco smoking [49], alcohol use [41, 49], binge drinking [41, 49], marijuana use [44, 49], inhalants use [49]; number of quits, life years gained [40, 46]; reductions in experimental smoking [50]; decreased smoking prevalence, delayed initiation of smoking, quality of life [43]; preventing methamphetamine use [30]; reductions in weekly smoking prevalence [51]; reductions in days detained, reductions in subsequent referrals [35]; reduced instances of crime [52]; increased condom or oral contraceptive use [34]; level of emotional distress, decreased externalising and internalising behaviours [47]. Some analyses broadened their measure of benefit and converted data into utility-based outcomes such as incremental cost effectiveness ratio (ICER) per DALY avoided [26], life years saved [37], QALYs gained [37], criminal activity free years (CAFYs) [42], and depression free days (DFDs) [45].

#### Costs and consequences

In the 17 CEAs and 1 CUA included in this review, the perspective employed varied from a societal perspective [26, 34, 39, 41, 42, 44, 45, 49], one of which included a government perspective [26]; a state government perspective [52]; a payer perspective to represent costs to the community [48]; a public sector perspective [43, 51]; a healthcare [50] or public health perspective [46]; to narrower perspectives such as an employer [30] or program perspective [47], with two analyses taking a single school perspective [37, 40]. Choice of perspective impacts on the number of costs and consequences that can be included in an analysis, but even when a broader perspective was chosen, the analysis wasn’t always applied to a broader population. Sheidow et al. [48], for example, chose a payer perspective and collected comprehensive program, court and treatment costs. However, despite acknowledging personal, social and economic costs across multiple service sectors, the analysis was applied only to individual participants in one county.

## Discussion

The purpose of this systematic review was to identify and critique full economic evaluations of interventions for high risk young people to inform the design of future economic evaluations of such programs. The 29 articles that met the criteria for inclusion in this review demonstrate a paucity of quality economic evaluations conducted of interventions for at risk youth in general. Consistent with a current review, no economic evaluations have been conducted of a multi-component community-based program specifically for high risk young people [4], even though it has been argued that such interventions are likely to be most effective for this population [1, 4]. Existing economic evaluations of youth programs can, however, inform the methodology of future economic analyses of interventions for high risk young people. Even though the sample populations of the studies under review may not all have been high risk young people, the domains of risk confronted by many at risk youth are similar. Most importantly for the young, high risk population, the methodology employed in economic evaluations of such interventions needs to address their complex aetiology.

### General methodological characteristics

What is striking about the 29 economic analyses included in this review is the heterogeneity of approaches to economic evaluation represented. This is unsurprising, given that most were conducted retrospectively and had potentially not planned to conduct an economic evaluation. The results described above document the different analytic characteristics of the studies and the economic analyses conducted of them. Often the analyst is unable to predetermine the type of analysis required as this will depend on the results of the clinical trial or the quality or availability of the data. As some studies demonstrated, different approaches are sometimes employed together in an attempt to better address the question at hand [30, 34, 26]. Alternatively, the decision maker may determine the choice of economic evaluation and narrow the perspective to address only their concerns or interests [35]. All or any of these considerations will impact on the choice of analysis and the way in which the cost and consequence data is measured and valued.

### Methodological challenges

As the results of the review demonstrate, there are three main challenges when conducting economic analyses of public health programs for at risk youth:

### Attribution of effects

Study design is a fundamental consideration when attributing causality of an impact to a public health intervention. Ideally, economic evaluations should be an integral part of the planning process of any intervention, allowing for prospective costing data and impact data collection. This is particularly important for interventions designed to address the complex needs of high risk young people. Inclusion of a control group and longitudinal rather than cross-sectional data collection facilitates the attribution of causality to the program [22, 25]. While RCTs may not always be ethically appropriate for a high-risk population, where a no treatment control group is preferred and matched control groups are difficult to source, study designs like multiple baseline design can provide a partial solution to this problem, particularly for a program implemented in various locations.

Complex health and social problems require complex interventions that also require appropriate evaluation designs [53]. Economic evaluations of these interventions also need to capture this complexity. Most interventions and their evaluations included in this review addressed too few domains of risk, despite the mutually reinforcing nature of these domains. Interventions for at-risk youth that focus on a single outcome, as was the case with many CEAs, is contrary to the nature of the treatment, which will have multiple effects on clients and their communities [44, 54]. Quite a number of economic analyses suffered from small [24, 44, 45, 47, 48] or non-generalisable samples [22, 28, 35, 48, 50, 54, 55], missing sample data [37, 42, 50], and poor quality or lack of evidence-based evaluations [43, 50]. This requires assumptions to be made about effect or impact. Sheidow et al. [48] addressed the problem of a small heterogeneous sample by averaging data rather than using individual data; a problem better addressed by larger sample size. Similarly, a lack of costing data means estimations must be made about costs [34]. Limited evidence of effect in program evaluation data or limited costing data can result in an under or over-estimation of the impacts of a program, impacting on the quality and precision of the economic analysis. While modelled parameters can be used to address missing or incomplete data, often the results of such an analysis can only be suggestive of actual impact.

The time frames used to evaluate interventions are another source of weakness for economic evaluations. Only eleven economic evaluations utilised data from follow-up of greater duration than 12 months [22–25, 30, 32, 33, 46, 49, 50], making it difficult to have confidence that an intervention would remain cost effective or cost beneficial in the long-term. For example, in an analysis of a smoking prevention intervention, without real data or longer term follow-up, there is no evidence to determine whether an intervention delayed or prevented smoking uptake [43], or whether cessation or any other behaviour of interest is maintained into the future [51]. Extrapolating effects into the future is particularly important in a youthful population. Youth is a time of transition and development, so program evaluations should strive to measure costs and outcomes across all time frames: short; medium and long term.

### Measuring and valuing outcomes

This review identified 29 empirical studies employing 32 economic evaluations comprising 13 CBAs (41%), 17 CEAs (53%), a CUA (3%) and a SROI (3%). As a consequence, outcomes in the economic evaluations varied: CEAs were based on a great diversity of outcomes, sometimes recorded in natural units such as number of incidences of substance abuse [48]; initiation of tobacco use, instances of delinquent behaviour [33]; number of (established) smokers prevented [31, 37]; days of abstinence, and so on (see Results 2b. above). Some analyses converted data into utility-based outcomes such as ICER per DALY avoided [26], life years saved [37], QALYs gained [37], criminal activity free years (CAFYs) [42], and depression free days (DFDs) [45].

Given the broader perspective usually associated with a CBA, in the 13 included CBAs and SROI, the number of outcomes measured varied from one to 19. However, for the nine analyses conducted from a societal perspective [22–25, 29, 31, 33, 34], the broadest and most comprehensive perspective, only four measured and valued more than three outcomes [22, 24, 25, 29]. Outcomes varied considerably across the CBAs and SROI based on the domain of risk being addressed by the intervention or the outcome of interest (See Results 1b above).

### Identifying costs and consequences

The perspective chosen in an economic analysis influences the types of costs and consequences included in the evaluation. Economic evaluations included in this review were conducted from a number of perspectives ranging from a broad societal perspective to employer, government, public sector, healthcare or even a single school perspective. Some incorporated multiple perspectives. A broader societal perspective in a CBA allows intersectoral outcomes to be included, thus enabling a more comprehensive economic evaluation. For example, French et al. [29] monetised 19 different outcomes from a broad range of domains such as health services utilization, substance abuse treatment utilization, education and employment and criminal activity. Kuklinski et al. [33] also included tangible costs and consequences from a broad range of sectors in their CBA such as increased earnings, decreased medical expenditure, reduced criminal justice system costs, as well as intangible effects such as pain and suffering and quality of life. From a single outcome of teenage births averted, Rosenthal et al. [24] monetised numerous benefits related to increased productivity and earnings, reduced public assistance, reduced incarceration, improvements in educational and employment opportunities, decreased social and economic support needs, improved personal motivation, and improved peer group influence.

Other economic evaluations limited the considered costs and consequences by using a narrower perspective. For example, Guyll et al. [30]. conducted a CBA and acknowledged the full range of benefits of youth programs, but then used a narrower employer perspective that excluded many potential benefits from being included in the analysis. Sheidow et al. [48] acknowledged the limitations of a CEA of an intervention that potentially has benefits across multiple domains; in these circumstances a CBA was suggested as a more appropriate form of economic analysis for its ability to include a range of benefits. 

### Implications for choice of analysis (CBA v CEA)

Despite acknowledging the limitations of their analysis, Sheidow et al. [48] conducted a CEA based on the costs and consequences of substance abuse and crime, which produced complicated results that were unlikely to optimally inform policy in the area. Two other multi-program CEAs demonstrated the difficulty associated with a CEA in a multiple domain context. In the French et al. [44] CEA, none of the three more costly interventions were more effective than the usual care psycho-educational group program for marijuana use or delinquency outcomes, so usual care was the most cost effective intervention. Similarly, as was the case with the Swisher CEA [49] where, after 2 years, the only significant effect of the multiple substance program was fewer female smokers in the intervention group, this program becomes the most cost effective, regardless of cost. French et al. [44] raise the methodological difficulty of more complicated CEAs that include multiple outcomes, stating that cost-effectiveness ratios for multiple outcomes can produce conflicting implications. CBA measures and values the outcomes of a public health program across numerous sectors including employment, family, education or the criminal justice system. CBA provides an estimate of the value of the resources used by a program compared to the value of the resources the program might save or generate [16]. In reality, often a CBA will compare only those costs and consequences that can readily be valued in monetary terms. In contrast, however, CEAs and CUAs assume that one program alternative will be most preferred, regardless of net benefit. Therefore, a program may be adopted which involves a net cost; that is, it does not generate benefits that exceed cost [16]. Under these circumstances, society is better off by rejecting the program – an outcome that would be highlighted with a CBA. Despite some analysts objecting to the monetisation of health related benefits needed for a CBA, when considering the shortfalls associated with CEAs of complex interventions such as those mentioned above, CBA seems a preferable and more appropriate approach to economic evaluation of interventions for at-risk youth [44, 54, 56].

### Limitations

A limitation of this systematic literature review was that despite a rigorous literature search conducted by an accredited librarian, many relevant references were not identified. Of the 29 articles that met the inclusion criteria, 17 were identified via grey literature or hand search, so there is the possibility that not every economic evaluation of an intervention for at risk young people will have been captured. Others have referred to similar difficulties sourcing studies when conducting literature searches of economic evaluations [53, 57].

It is also possible that identified economic evaluations may have been inaccurately classified, however, given the clearly articulated exclusion criteria and the inter-rater agreement on classification and quality assessment, this is unlikely.

A further limitation is the use of the quality assessment rating scale [21]. Despite it being based on the Drummond checklist, a well-known economic tool, it is not a standardised rating scale.

### Recommendations for future research

This review has identified that CBAs are most appropriate for capturing the multiple impacts interventions for high risk young people have on participants and society, including the consequential downstream benefits. A societal perspective is the preferred approach for CBA because it allows a broader, more comprehensive analysis. In addition, from a theoretical perspective, CBA is consistent with welfare economics, where all costs and benefits are considered, regardless of who benefits [58]. Many of the methodological challenges highlighted by this review could potentially have been addressed if the economic analyses were conducted prospectively and economists were involved in discussions of study design, and cost and outcome data collection. Prospective long-term data collection and a strong study design that incorporates a control group contribute to the quality of economic evaluation. Where RCTs are not possible, other study designs may provide a partial solution to this problem. In addition, extrapolation of costs and impacts into the future is important for youthful populations in order to account for the time lag in effect of the many benefits arising from interventions for high risk young people. Interventions for high risk young people should target multiple domains of benefit and economic evaluations of such interventions need to capture this complexity [14, 15]. However, techniques for monetization of benefits need to be developed and agreed upon.

## Conclusion

This is the first systematic review of economic evaluations conducted of interventions for high risk young people. A number of methodological challenges were identified, highlighting the need for not only more economic evaluations of interventions that address multiple domains of risk, but better quality economic evaluations. Rigorous economic evaluation of interventions for high risk young people is particularly important given the necessity for more complex interventions designed to address numerous domains of risk. As the above review has demonstrated, greater impacts and more long term benefits have been shown to result from prevention programs implemented early in the adolescent pathway [8, 12, 30, 59]. There is a particular need for economic evaluations of multi-component programs for high risk young people, given this review found none currently exist. A CBA is more likely to capture the dynamic nature of this population and allow for the incorporation of broader intersectoral outcomes. From a policy perspective, there is a need for more high quality economic evaluations to better inform decisions about competing uses of limited resources.

## Additional files

Additional file 1: Search strings used in search strategy (Embase, Medline & PsychInfo). (DOCX 19 kb)

Additional file 2: Summary table of included economic evaluations. (DOCX 67 kb)

Additional file 3: Quality assessment of economic evaluations using the Drummond checklist. (DOCX 18 kb)

## Abbreviations

ACRA: Adolescent community reinforcement approach
APO: Australian policy online; economic evaluation
BCR: Benefit cost ratio
CAEPR: Centre for aboriginal economic policy research
CAFY: Criminal activity free year
CBA: Cost-benefit analysis
CBI: Cognitive behavioural intervention
CBT: Cognitive behaviour therapy
CEA: Cost-effectiveness analysis
CEAC: Cost-effectiveness acceptability curve
CUA: Cost-utility analysis
CYT: Cannabis youth treatment study
DALY: Disability adjusted life year
DFD: Depression free day
FC: Family court
FFT: Family first therapy
FSN: Family support network
ICER: Incremental cost-effectiveness ratio
I-LST: Infused life skills training
ISFP: Iowa strengthening families program
ISM: Intensive supervision and monitoring
JDC: Juvenile drug court
LST: Life skills training
LYS: Life year saved
MARS: Motivating adolescents to reduce sexual risk
MDFT: Multidimensional family therapy
MET: Motivational enhancement therapy
MST: Multisystemic Therapy
NICE: National institute for health and care excellence
QALY: Quality adjusted life year
RCT: Randomised controlled trial
SROI: Social return on investment
STI: Sexually transmitted infection
TAU: Treatment as usual
TNT: Towards no tobacco use
WSIPP: Washington state institute for public policy
