# Supplementary material for: Systematic review of economic evaluations of interventions for high risk young people
Source: BMC Health Serv Res. 2018 Aug 23;18:660. doi: 10.1186/s12913-018-3450-x (PMC6108123; doi:10.1186/s12913-018-3450-x)
Supplement: Supplementary file 1 — Search strings used in search strategy (Embase, Medline & PsychInfo). (DOCX 19 kb) [file 12913_2018_3450_MOESM1_ESM.docx]

Additional file 1: Sample search strings for Embase, Medline & PsychInfo

| **EMBASE** | | |
| --- | --- | --- |
| **#** | **Searches** | **Results** |
| 1 | exp *young adult/ | 1959 |
| 2 | exp *juvenile/ | 311486 |
| 3 | exp *adolescence/ | 35143 |
| 4 | exp *adolescent/ | 40874 |
| 5 | teen*.tw. | 31382 |
| 6 | youth.tw. | 51211 |
| 7 | or/1-6 | 401155 |
| 8 | exp mental disease/ | 1800767 |
| 9 | exp drug abuse/ | 67574 |
| 10 | exp substance abuse/ or exp drug dependence/ | 119818 |
| 11 | exp alcohol abuse/ | 28871 |
| 12 | exp alcohol consumption/ | 88708 |
| 13 | exp juvenile delinquency/ | 8526 |
| 14 | exp intellectual impairment/ | 413271 |
| 15 | exp socioeconomics/ | 202680 |
| 16 | exp child health care/ | 71438 |
| 17 | health service/ | 143568 |
| 18 | exp health education/ | 259622 |
| 19 | exp health education/ or exp health promotion/ | 259622 |
| 20 | exp health program/ | 99186 |
| 21 | exp school health service/ | 20004 |
| 22 | exp health care policy/ | 157403 |
| 23 | exp family/ or exp family relation/ | 428391 |
| 24 | exp employment/ | 61762 |
| 25 | exp crime/ | 75050 |
| 26 | or/8-25 | 2996577 |
| 27 | randomized controlled trial/ | 394033 |
| 28 | exp pilot study/ | 93877 |
| 29 | exp prospective study/ | 321316 |
| 30 | exp program development/ | 19547 |
| 31 | youth program*.tw. | 208 |
| 32 | exp intervention study/ | 26765 |
| 33 | or/27-32 | 796752 |
| 34 | exp "cost benefit analysis"/ | 70619 |
| 35 | exp "cost effectiveness analysis"/ | 111875 |
| 36 | exp "health care cost"/ or exp "cost utility analysis"/ or exp economics/ | 438249 |
| 37 | exp health economics/ | 681566 |
| 38 | exp economic evaluation/ | 237981 |
| 39 | economic analysis.tw. | 4802 |
| 40 | or/34-39 | 815521 |
| 41 | 7 and 26 and 33 and 40 | 275 |
| 42 | limit 41 to (human and english language and yr="2000 -Current") | 234 |
| 43 | limit 42 to (embase and (article or journal or report or "review")) | 142 |
| 44 | remove duplicates from 43 | 141 |
| 45 | from 44 keep 1-141 | 141 |

| **MEDLINE** | | |
| --- | --- | --- |
| **#** | **Searches** | **Results** |
| 1 | exp *Young Adult/ | 77 |
| 2 | exp *Adolescent/ | 5527 |
| 3 | exp *Adolescent Behavior/ or exp *Adolescent Health Services/ | 19071 |
| 4 | teen*.tw. | 21701 |
| 5 | juvenile.tw. | 53494 |
| 6 | youth.tw. | 35496 |
| 7 | or/1-6 | 124477 |
| 8 | exp Mental Disorders/ | 1017282 |
| 9 | exp Substance-Related Disorders/ | 237472 |
| 10 | exp Juvenile Delinquency/ | 7616 |
| 11 | exp Intellectual Disability/ | 84774 |
| 12 | exp Socioeconomic Factors/ | 367438 |
| 13 | exp "Health Services Needs and Demand"/ | 52074 |
| 14 | exp Health Education/ | 142906 |
| 15 | exp Health Promotion/ | 59993 |
| 16 | exp School Health Services/ | 19956 |
| 17 | exp Health Policy/ | 87840 |
| 18 | exp Family/ or exp Family Relations/ | 256318 |
| 19 | exp Employment/ | 68951 |
| 20 | exp Crime/ | 112343 |
| 21 | or/8-20 | 1862916 |
| 22 | exp Prospective Studies/ | 402411 |
| 23 | exp Randomized Controlled Trials as Topic/ | 101650 |
| 24 | exp Pilot Projects/ | 90816 |
| 25 | exp Program Development/ | 24217 |
| 26 | exp Intervention Studies/ | 0 |
| 27 | youth programs.tw. | 116 |
| 28 | or/22-27 | 603446 |
| 29 | exp Cost-Benefit Analysis/ | 64140 |
| 30 | exp Economics/ | 518218 |
| 31 | exp Health Care Costs/ | 50656 |
| 32 | economic evaluation.mp. | 5329 |
| 33 | economic analysis.mp. | 3042 |
| 34 | or/29-33 | 519916 |
| 35 | 7 and 21 and 28 and 34 | 115 |
| 36 | limit 35 to (english language and humans and yr="2000 -Current") | 66 |
| 37 | limit 36 to (evaluation studies or government publications or journal article or meta analysis or randomized controlled trial or "review") | 63 |
| 38 | remove duplicates from 37 | 63 |
| 39 | from 38 keep 1-63 | 63 |

| **PsycINFO** | | |
| --- | --- | --- |
| **#** | **Searches** | **Results** |
| 1 | young adult.tw. | 9894 |
| 2 | exp Adolescent Development/ | 39138 |
| 3 | youth.tw. | 66141 |
| 4 | teen*.tw. | 18712 |
| 5 | juvenile.tw. | 21640 |
| 6 | or/1-5 | 135751 |
| 7 | exp Mental Disorders/ | 488270 |
| 8 | exp Drug Abuse/ | 95469 |
| 9 | exp Alcohol Abuse/ | 42186 |
| 10 | substance abuse.tw. | 29932 |
| 11 | exp Juvenile Delinquency/ | 17122 |
| 12 | exp Intellectual Development Disorder/ or exp Cognitive Impairment/ | 67507 |
| 13 | exp Socioeconomic Status/ | 42389 |
| 14 | exp Health Care Services/ or exp Health Care Delivery/ | 110658 |
| 15 | exp Health Education/ | 15700 |
| 16 | exp Health Promotion/ | 18622 |
| 17 | "school health services".tw. | 112 |
| 18 | exp Dysfunctional Family/ or exp Family Relations/ or exp Family/ | 149608 |
| 19 | exp Employment Status/ | 16248 |
| 20 | exp Crime/ | 84721 |
| 21 | or/7-20 | 974775 |
| 22 | exp Intervention/ | 76412 |
| 23 | prospective stud*.tw. | 12350 |
| 24 | exp Program Development/ | 7816 |
| 25 | exp School Based Intervention/ | 12976 |
| 26 | exp Family Intervention/ | 2392 |
| 27 | pilot project*.tw. | 1484 |
| 28 | controlled stud*.tw. | 10143 |
| 29 | or/22-28 | 106410 |
| 30 | exp Health Care Costs/ or exp Economics/ | 27611 |
| 31 | "cost benefit analyis".tw. | 0 |
| 32 | "cost effectiveness analysis".tw. | 814 |
| 33 | "cost utility analysis".tw. | 193 |
| 34 | exp Health Care Economics/ | 579 |
| 35 | "economic evaluation".tw. | 940 |
| 36 | "economic analysis".tw. | 750 |
| 37 | or/30-36 | 28877 |
| 38 | 6 and 21 and 29 and 37 | 28 |
| 39 | limit 38 to (human and english language and yr="2000 -Current") | 28 |
| 40 | limit 39 to (journal article or reviews) | 12 |
| 41 | from 40 keep 1-12 | 12 |
