# Supplementary material for: Systematic review of economic evaluations of interventions for high risk young people
Source: BMC Health Serv Res. 2018 Aug 23;18:660. doi: 10.1186/s12913-018-3450-x (PMC6108123; doi:10.1186/s12913-018-3450-x)
Supplement: Supplementary file 2 — Summary table of included economic evaluations. (DOCX 67 kb) [file 12913_2018_3450_MOESM2_ESM.docx]

Additional file 2 Summary table of included economic evaluations

| **13 Cost benefit analyses and 1 social return on investment** | | | | | | |
| --- | --- | --- | --- | --- | --- | --- |
| **First author & year of publication (country)** | **Sample/setting** | **Study/Intervention** | **Domains of risk (Knight et al. 2015)** | **Outcomes in economic evaluation (EE)** | **EE type & perspective** | **Key study findings & methodological insights** |
| Belfield 2003 (US)[23] | 1138 youths, 8 high caseload agencies  across 6 states  10-17 yrs | Big Brothers Big Sisters (BBBS) one-on-one mentoring program- participants randomly assigned to program or waitlist (control group). Based on impact evaluation of BBBS by Grossman & Tierney (1998) | Education & employment  Substance use  Criminal activity  Violence | Reduced drug use  Increased educational attainment  Reduced criminal activity | Retrospective CBA  3 perspectives: youth participants; mentors; social/taxpayers | CBR reduced drug use 1.52:1;  CBR improved educational attainment 1.61:1;  CBR reduced criminal activity 0.97:1  Benefits not aggregated |
| Rosenthal 2009 (US)[24] | 50 teenagers per year (25 + 25 male) Greater New Britain, Connecticut.  12-18 yrs | Pathways neighbourhood-based program to prevent unintended pregnancies & promote youth development.  6 component program: i) education about family life, sex, & health; ii) academic support- weekly tutoring; iii) career & vocational preparation; iv) artistic expression; v) recreation; vi) physical & mental healthcare referrals. 7 year enrolment | Sexual behaviour  Education & employment  Mental health & wellbeing | Reductions in teenage births: | Retrospective CBA  Perspective: societal | Net Social Benefit calculated at 20, 25 & 30years  Total costs to society exceeded benefits by almost US$1600 (at 2006 prices) per teen at time of participation  Total social benefits would outweigh social costs by age 20.1 years  Small sample size and specific to one neighbourhood, so not necessarily generalizable to other contexts  No control group, so no way of a determining causal association between program & outcomes |
| Zerbe 2009 (US)[25] | 479 alumni out of care for between 1-13 yrs. 111 from Casey Foster Care (private) and two public foster care providers (126 & 242) in 2 states.  Adults who were in foster care between ages 14-18 | Casey Family Programs Foster Care services compared to two state services. Participants over 18 and out of foster care for over 1 year | Education & Employment  Homelessness  Mental Health & Wellbeing | Education  Employment  Finances  Marriage  Children  Social networks  Physical health  Mental health | Prospective CBA  Perspective: Societal | BCR 1.7:1  Difficult to get a prospective control group for this population-reason for study design  Causality not established, but Casey participants had been state service recipients previously, so similar population characteristics.  Propensity score adjustment used for differences between groups |
| SVA Consulting 2014 (Australia)[37] | 300 Indigenous participants living in Shepparton area of Victoria 12-25 yrs | Ganbina –School to work program for Indigenous students | Education & employment  Mental health & wellbeing  Criminal activity  Sexual behaviour | Education & employment Health & wellbeing  Criminal activity  Sexual behaviour | Retrospective Social Return On Investment (SROI)  Perspective: social | SROI 6.7:1  Indigenous only program limited to one area, so not likely to be generalisable |
| Access Economics 2008 (Australia)[36] | Youth from Interface municipalities (9) around Melbourne (32% of Melbourne’s population) 10-24 yrs | Interface-recommended programs to engage youth in education and address mental health | Education & employment Mental health & wellbeing | CBA-Improved productivity & earning capacity;  Reduced health system expenditure;  Value of statistical Life (VSL);  CEA-Decreases in burden of disease | Hypothetical CBA of proposed interventions 2009-2012  Perspective: societal & government  Comparator TAU | BCR 23.6:1 returns to society; 7.6:1 to govt in increased tax revenue (education)  Outcomes unknown for proposed interventions so 3 scenarios provided (worst, base, best)  Focussed on two outcomes only |
| Kuklinski 2012 (US)[33] | 4,407 students surveyed annually 2003-2008  10-14 yrs | RCT of Communities that Care (CTC) intervention in 24 communities in 7 states (Hawkins et al 2008) | Criminal activity  Substance use (tobacco only) | Delinquency  Tobacco smoking | Retrospective CBA  Perspective: participants, taxpayers & general public | BCR $5.30:1 - $10.23:1 depending on cost assumptions  Used benefits from Aos et al. 2004 (see above) to value crime outcomes for delinquent behaviour & cost of tobacco consumption. Lifetime impact  Only 2 outcomes measured, yet many more possible |
| Hoeflmayr 2008 (Germany)[39] | 2,142 adolescents elected to be non-smoking for 6 months  Hamburg, Berlin & Hanover  11-14 yrs | School-based tobacco prevention program-Smoke-free Class Competition (SFC)  Experimental group pre-selected from Hamburg & Berlin; control chosen at random from Hanover German component of program implemented in 20 European countries | Substance use (tobacco only) | No. of smokers prevented  Smoking status (4 week prevalence) assessed 1 month before competition; 1 month after & 1 year after | Retrospective CBA  Perspective: societal | CBR: 3.6:1  Uses actual cost rather than modelled data  Wang et al. (2001) progression model used  Outcome evaluation based on follow-up 12 months post program; not long enough to be sure prevention is sustained |
| Guyll 2011 (US)[30] | 1,125 6^th^ or 7^th^ grade students from 58 Iowa school districts  905 students at follow-up in grade 12  12/13 -17/18 yrs | RCTs of 3 preventive interventions **1.**Iowa Strengthening Families Program (ISFP);  **2.** Life Skills Training (LST);  **3**.(ISFP+LST) to delay or prevent the onset of substance use and delinquent behaviours  ITT approach used to calculate intervention effects | Substance use (methamphetamine only) | Preventing one 12 grade adolescent from using methamphetamine in past year | Retrospective CBA  Perspective: employer  (human capital approach- increased productivity; reduced absenteeism; healthcare costs, theft & turnover) | NPV: US$97,523 at time of intervention implementation (at 2009 prices)  BCRs:  1. 3.84:1;  2.19.04:1;  3.1.56:1  Only those in school to year 12 ; what about those not in school??  Benefits limited to market value of increased productivity associated with methamphetamine use prevention: decreased absenteeism, healthcare costs, theft, turnover & increased productivity. Data from US Dept of Health & Human Services, Bureau of Labor Statistics, Association of Fraud Examiners & surveys of personnel professionals used to determine costs.  Many more potential benefits from interventions not analysed. |
| French 2003 (US)[29] | 600 cannabis using adolescents and their families in the Cannabis Youth Treatment study, a collaboration between Center for Substance Treatment, two treatment providers in Illinois & Florida, & two major medical centres in Connecticut & Philadelphia  12-17 yrs | Two RCTs of five short term (<90 days) outpatient interventions   1. Motivational enhancement treatment/cognitive behaviour therapy (MET/CBT5) -5 sessions CBT 2. MET/CBT12-12 sessions CBT 3. Family support network (FSN); 4. Adolescent community reinforcement approach (ACRA); 5. Multidimensional family therapy (MDFT)   Trials:  1.Incremental (1.,2.& 3.);  2.Alternative (1.,4.& 5.) | Substance use (cannabis) | 19 different outcomes monetised from broad domains such as health services utilisation, substance abuse treatment utilisation, education & employment, & criminal activity | Retrospective CBA  Perspective: societal | Net economic benefits positive for three of the five interventions: MET/CBT5, MET/CBT12 & FSN  90% follow-up in quarterly waves over 12 mths  “No treatment” control group needed  self report data used |
| Robertson 2001 (US)[35] | 293 offenders on probation or parole in Mississippi in 3 participating county youth courts  11-17 yrs | Community-based intensive supervision & monitoring (ISM) & cognitive behavioural therapy intervention (CBTI) (6mths) vs control group experiencing standard probation & parole via local court authorities. Each county court adopted one of the interventions or the control  18 month follow up | Criminal activity | Reductions in days detained  Reductions in subsequent referrals | Prospective CBA  Perspective: local justice system | CBTI group had significantly lower mean expenditures on the justice system court referrals and days of detention, whereas the ISM group had slightly greater mean expenditures than the control group.  CBR for CBTI 1.96:1  Quasi-experimental design;  Direct, short run marginal benefits & costs only: costs avoided for court referrals and days of detention;  Narrow perspective and findings unlikely to be generalisable  No societal benefits included  More outcomes and long term data needed |
| Cartwright 2009 (US)[27] | Demographic economic model based on evidence-based outcomes of MST intervention  12-17 yrs | Multisystemic Therapy (MST) for serious juvenile offenders  2 demographic economic models of expanding implementation of MST | Criminal activity | Reduced residential placements and re-arrests | Retrospective CBA  Perspective: taxpayers & victims  Two scenarios using model that projects youth in residential custody to illustrate effect of MST | First scenario entails increased financing (3%/year) of MST with treated youth replaced by another delinquent. Economic benefit taken from a WSIPP study (Aos et al. 2004) comprising a taxpayer benefit of US$7238 & a non-taxpayer benefit US$7758. After adjustment to 2007 dollars, economic benefit is $16,226.  CBR: 1.42:1  Alternative scenario & much less costly option: increase number treated to1,000 and maintain funding for 10 years. Success reduces the number of residents and subsequent costs over 10 years. This approach resulted in reductions in residential incarceration of African American youth and white youth of 25% and 34%, respectively.  CBR 1.31:1  Based on clinical trials of short duration; doesn’t include all benefits, only cost avoidance for crime, so benefit estimation is biased downward. The model extrapolated high levels of residential care from fairly limited data collected in 2001 & 2003. |
| Klietz 2010 (US)[32] | 176 serious and violent juvenile offenders participating in Multisystemic Therapy (MST)  Average age at intervention 14.5 yrs | Clinical trial of MST versus individual therapy (IT)  MST is a family and community-based treatment with evidence of long term impact on criminal activity (Schaeffer & Borduin 2005) | Criminal activity | No. of arrests per participant for different categories of crime: murder/manslaughter; sexual; robbery; aggravated assault; property; drug. | Retrospective CBA  Perspective: taxpayer & victim | Benefits of MST are reductions in: 1.taxpayer costs; 2.tangible costs; & 3.Intangible costs to crime victims  BCR 9.21:1 - 23.59:1  Washington State Institute for Public Policy (WSIPP) model adapted to long term (13.7 yrs) follow-up of clinical trial[63]  Improvement on WSIPP CBA of MST by using continuous rather than dichotomous outcome measure of recidivism for intended MST population, serious juvenile offenders |
| Wang 2000 (US)[34] | 345 high school students from 10 schools (5 randomised to intervention; 5 to control) in northern California & the same for 10 schools in SE Texas  12-18 yrs | Safer Choices, a school-based HIV, other STDs & teen pregnancy prevention intervention for high school students | Sexual behaviour | Increase in condom use (STDs & pregnancies averted); | Retrospective CBA  Perspective: societal | Net benefit US$174,276 (at 1994 prices); BCR: 2.65:1  Data from 7 month follow up only;  Some cost data outdated or missing, so estimated: direct costs of program including materials such as condoms and oral contraceptives; medical and social costs averted.  Social costs included foregone wages in lost productivity for HIV infection, and costs per case of childbearing for an 18 year old, which included earnings related outcomes and public assistance  assumptions about heterosexual sex only  No directly measured data available so modelling used for STDs & pregnancies averted based on literature. |
| Dealy 2013 (US)[28] | 264 justice-involved youth predominantly Hispanic sample from South Western US;  206 completed 3 mth follow-up  14-18 yrs | Motivating Adolescents to Reduce Sexual Risk (MARS) program to change risky sexual behaviour  Participants randomly assigned to  1.sexual risk reduction intervention (SRRI);  2.SRRI + alcohol risk reduction;  3. SRRI + alcohol risk reduction + marijuana risk | Sexual behaviour | Averted STIs | Preliminary CBA of short term outcomes  Perspective not stated, but healthcare perspective inferred | Reduction in direct medical costs  CBR for averting STIs (chlamydia, gonorrhoea) 2.08:1; with inclusion of viral STIs 2.68:1  Participants WTP for averted STIs increased after MARS intervention.  Preliminary, so 3 month follow-up only  Rigorous costing conducting conducted including all resources used, infrastructure, labour & training. Pre post model incorporating changes in risky sexual behaviour & changes in probability of infection employed.  More outcomes could have been evaluated  Not representative population; financial incentives for completing follow up at 3 months |
| **17 Cost effectiveness analyses and 1 cost utility analysis** | | | | | | |
| Sheidow 2012 (US)[51] | 161 juvenile offenders on probation approved for entry to a drug court program (128 included in final evaluation). Youth with substance use disorders only. 12-17 years | RCT Juvenile Drug Court program + evidence-based treatments compared with Family Court + community services [TAU]; JDC + community services; JDC+MST; & JDC+ MST+CM  (Henggeler 2006) | Criminal activity Substance use | Mean number of incidences reduced; e.g. days of marijuana use, poly-drug use, alcohol use, and heavy alcohol use; SRD status offenses, theft, crimes against persons. Baseline & 12 mths | Retrospective CEA  Comparator: TAU | Integration of evidence-based treatments into JDC cost- effective in decreasing substance use and delinquent behaviour.  CE tended to improve with increased intensity of interventions.  FC +community services most cost-effective in reducing marijuana use & theft (on average) of four interventions.  JDC + community services efficient in reducing delinquent behaviour & marijuana use.  JDC + MST efficient for reducing all outcomes except alcohol use & most cost-effective for reducing status offences  & crimes against persons.  JDC + MST + CM efficient in reducing all outcomes & most cost-effective in reducing poly drug use, alcohol use,  & heavy alcohol use.  Effect measured after only 12 months.  Complicated findings due to use of CEA  FC most cost-effective due to low cost not clinical effectiveness.  JDC three times more costly than FC, but more cost effective for criminal behaviour.  Variation in how JDC operates so costs & results possibly not generalizable.  Suggest CBA may be preferable so more domains can be monetised. |
| Access Economics 2008 (Australia)[36] | Youth from Interface municipalities (9) around Melbourne (32% of Melbourne’s population) 10-24 yrs | Interface-recommended programs to engage youth in education and address mental health | Education & employment Mental health & wellbeing | CEA-Decreases in burden of disease | Hypothetical CEA of proposed interventions 2009-2012  Perspective: societal & government  Comparator TAU | CEA: mental health ICER AU$3922 (at 2009 prices)/DALY avoided (base case figures)  Outcomes unknown for proposed interventions so 3 scenarios provided (worst, base, best) |
| Wang 2001 (US)[40] | 1234 Yr 7 students participated in intervention;  770 year 9 students participated in 2 year follow-up  1956 students recruited as control group;  1565 participated in 2 year follow-up 13-15 yrs | Towards No Tobacco (TNT) program to counteract influences to use tobacco. Participants randomly allocated to 4 curriculum groups: 1.normative social influence; 2. Physical consequences; 3.informational social influences; 4.combination OR 5.‘usual care’ control group | Substance use (tobacco only) | Numbers of established smokers prevented | Retrospective CUA  Comparator: usual care group  Perspective: school | At an estimated cost of US$16,403 (at 1990 prices), 34.9 students were prevented from becoming smokers  US$13,316 (at 1990 costs) per LY saved & US$8482 (at 1990 costs) per QALY saved (incl. medical costs)  US$481 - US$2770 per LY saved & US$306 – US$448 per QALY saved (at 1990 costs) (excl. medical costs)  Costs estimated  No. of smokers prevented modelled rather than measured;  Smoking progression model only for ages 14-26;  Little or no data to support progression for each age interval, so assumptions made. |
| Ross 2006 (US)[49] | 7725 students in Years 7, 8, 11 & 12  Tuscon, Arizona  13-18 yrs | Full Court Press, comprehensive, community-based adolescent tobacco use prevention program (1996-2001) | Substance use  (tobacco only) | Number of adolescents who quit smoking  Life years gained | Retrospective CEA  Perspective: public health | Costs per quit $3789  Costs per discounted life year gained $3942  No control group; attempt made to compensate for this using potential impact of cigarette price changes and the presence of other tobacco control programmes.  Some data extrapolated |
| Dino 2008 (US)[43] | 566 youths who smoked 5+ cigarettes a day  Florida - statewide  14-19 yrs | Not-On-Tobacco (N-O-T) teen smoking cessation intervention – whole health approach vs Brief Intervention (BI) typical school  (Dino et al. 2001) | Substance use  (tobacco only) | Life years saved | Retrospective CEA  Perspective: school-based  Comparator: Brief Intervention | 5.1 life years saved for quitters; 3.7 life years for reducers  Best case: ICER $273.60 per DLY saved. Worst case: ICER $1028.90 per DLY saved. Base case ICER: $442.65 per DLY saved  Markov model used to explain stage progression of smoking cessation among participants from age of 17-25 |
| Vijgen 2008  (Netherlands)[53] | 51 classes from 32 schools: intervention group (social influence program-SI)  67 classes from 20 schools: control group  14-15 yrs | Randomized trial  Schools randomly allocated to 2 interventions & control group: 1. SI program; 2. SI program + boosters & 3. control group (Dijkstra et al. 1999)  6mth follow-up (n=4060); 12mth follow-up (n=3653); 18mth follow-up (n= 3104) | Substance use (tobacco only) | Experimental (not regular) smoking  Have you smoked during the last month? Doesn’t discriminate between daily and occasional smoking | Retrospective CEA  Perspective: healthcare (all related and unrelated future health care costs and effects included due to longer life years)  Comparator: no intervention | €19,900 (at 2004 prices) per QALY gained.  Greatest health gains from reductions in daily smoking, so long-run health gains difficult to measure  Chronic Disease Measure (CDM) used to extrapolate reductions in no. of smokers over 100 years. Greatest health gains 60-100yrs. Absence of evidence on effectiveness, assumptions used. Outcomes modelled not measured.  Setting specific results, so not generalisable |
| Jit 2009 (UK)[46] | School-based smoking prevention programs  International  11-23 yrs | Cost-effectiveness model of school-based interventions to prevent the uptake of smoking among children and young people | Substance use (tobacco only) | Decrease in smoking prevalence  Delayed initiation of smoking  QoL | Retrospective modelled CEA  Perspective:  public sector  Comparator:  no intervention | Different effect models used to calculate CE (at 2003 prices):   1. Delay in effect with age: £15,900 per QALY gained 2. Decrease in effect with age: £2370 per QALY gained 3. Delay in effect with time: £45,700 per QALY gained 4. Decrease in effect with time: £1,780 per QALY gained   Outcomes modelled not measured  Lack of long-term evidence  Difficult to determine whether attenuation effects due to age of participants or time since intervention |
| Hollingworth 2012 (UK)[54] | 10,730 Year 8 students from 59 schools in South East Wales and West England  12-13 yrs | Cluster RCT of peer led smoking prevention program (ASSIST) in schools (29 control, 30 intervention) | Substance use (tobacco only) | Weekly smoking prevalence at 2 year follow up: 2.1% reduction | Prospective CEA  Comparator: no intervention  Perspective: public sector | ASSIST effective in reducing smoking prevalence, costing approximately UK£1500 (at 2008 prices) per child not smoking at 2 years.  Based on assumptions about the extent to which reductions in adolescent smoking prevalence led to lower prevalence or earlier cessation in the adult population. Only 1 year analysis: no knowledge of future maintenance or behaviours |
| Swisher 2004 (US)[52] | 732 students from  9 low SES rural schools in Pennsylvania (3 schools in each condition)  12-13 yrs | Prospective cluster RCT of 2 approaches to Life Skills Training (LST)[64]  2 year follow-up  LST n=234  I-LST n=297  No treatment control n=201 | Substance use (including tobacco) | Alcohol use  Binge drinking  Marijuana use  Inhalants  Tobacco smoking | Retrospective CEA  Perspective: Public sector cost  Comparator: No intervention | No statistically significant impact on males (54% of sample) after years 1 & 2  LST females after year 1: reduced smoking, alcohol use, binge drinking, marijuana use & inhalant use  I-LST females after 1 year: reduced smoking, binge drinking, marijuana use. I-LST females after 2 years: reduced smoking only.  Incremental cost per 7^th^ grade student: I-LST US$129.11; LST US$95.65  Incremental cost per 8^th^ grade student: I-LST US$116.69; LST US$62.16  (at 2000 prices)  Some details re trial & outcomes not reported  Self report data used  Only additional costs (training, materials & teachers salaries) required to implement program calculated based on assumption that schools already have required personnel and infrastructure.  No discounting |
| French 2008 (US)[47] | 114 youths with substance abuse disorder Albuquerque, New Mexico –  13-17 yrs | RCT  Four interventions:  1. Individual CBT,  2. Functional Family Therapy,  3. Integrative individual & Functional Family Therapy (CBT & FFT),  4. Psychoed group intervention.  Follow-up at 4mths & 7mths post treatment | Substance use (illicit only) | Rate of marijuana use (1% point reduction in days of marijuana use);  Reduction in juvenile delinquency (one unit reduction in delinquency score) | Retrospective CEA  Not an incremental CEA: comparison of mean values for treatment cost and effectiveness for each intervention to quantify relative effectiveness  Perspective: societal | CEA for each intervention – disaggregated  FFT showed significant reduction in cannabis use at 4 months, but no significant differences in cannabis use or delinquency at 7 months, so the least costly intervention by default became the most cost effective  Interventions for at-risk youth that focus on a single outcome are contrary to the nature of the treatment which will have multiple effects on clients and their communities  CE ratios for various outcomes can produce conflicting implications (Sindelar et al.2004)[59]  CBA preferable approach.  Small sample size  Comprehensive costing conducted using DATCAP |
| Dennis 2004 (US)[42] | 600 cannabis using adolescents and their families in the Cannabis Youth Treatment study, a collaboration between Center for Substance Treatment, two treatment providers in Illinois & Florida, & two major medical centers in Connecticut & Philadelphia  12-17 yrs | Two RCTs of five short term (<90 days) outpatient interventions   1. Motivational enhancement treatment/cognitive behaviour therapy (MET/CBT5) -5 sessions CBT 2. MET/CBT12-12 sessions CBT 3. Family support network (FSN); 4. Adolescent community reinforcement approach (ACRA); 5. Multidimensional family therapy (MDFT)   Trials:  1.Incremental (1.,2.& 3.);  2.Alternative (1.,4.& 5.) | Substance use (cannabis) | Days of abstinence;  Per cent of adolescents in recovery (no substance use problems and living in community) | Retrospective CEA  Comparators: other treatments  Perspective: societal | Cost-effectiveness results:  In Trial 1, MET/ CBT5 and to a lesser extent MET/CBT12 were more cost effective than FSN.  In Trial 2, ACRA and to a lesser extent MET/CBT5 were more cost effective than MDFT.  Similar clinical outcomes achieved sites & conditions in the 2 RCTs, but moderate to large differences in cost effectiveness. Significant cost differences by condition in each of the 4 sites. |
| Guyll 2011 (US)[30] | 1,125 6^th^ or 7^th^ grade students from 58 Iowa school districts  905 students at follow-up in grade 12  12/13 -17/18 yrs | RCTs of 3 preventive interventions **1.**Iowa Strengthening Families Program (ISFP);  **2.** Life Skills Training (LST);  **3**.(ISFP+LST) to delay or prevent the onset of substance use and delinquent behaviours | Substance use (methamphetamine only) | Preventing one 12 grade adolescent from using methamphetamine in past year | Retrospective CEA: Comparators: 3 interventions Perspective: employer | ICERs (at 2006 prices):  1. US$25,385;  2. US$5122;  3. US$62,697  Only those in school to year 12: What about those not in school??  Many more potential benefits from interventions not analysed. |
| Ingels 2013 (US)[44] | 473 rural African American adolescents and their primary caregivers  15-16 yrs | RCT Strong African American Families-teen program (SAAF-T) vs Attention Control Intervention (ACI) (comparator)  Data collected at 3 month pre-test  & 15-18 months post intervention | Substance use (alcohol only) | Self report data on how often one drink consumed in past 3 months (use) & how often three drinks or more consumed at once in past three months (binge) | Retrospective CEA  Comparator: Attention Control Intervention (ACI)  Perspective: societal | ICER for SAAF-T relative to ACI US$50 (at 2009 prices) for each reduced episode of alcohol use & US$123 (at 2009 prices) for each episode of reduced binge drinking  CEACs used to illustrate probability of cost effectiveness for both alcohol use and binge drinking episodes prevented over a range of willingness to pay values.  At a threshold of US$100 and US$440, there is a 90% probability of SAAF-T being cost effective compared to ACI for reductions in alcohol use and binge drinking episodes, respectively.  Rigorous costing based on prospective costing of final two phases of intervention (incl. implementation participant & donated resources); remaining costs estimated through multiple imputation |
| Schawo 2012  (Netherlands)[45] | Markov model to assess CE of preventing juvenile delinquency  Youth (hypothetical) | Illustrative use of systemic intervention Functional Family Therapy (FFT) compared with TAU to demonstrate Markov model with non-health measure-CAFYs | Criminal activity | Criminal activity free years (CAFYs) | Retrospective modelled CEA  Comparator: TAU  Perspective: societal | Illustrative model suggests dominance of FFT compared to TAU for juvenile delinquency.  Interesting alternative to a health measure used to evaluate interventions that are not primarily aimed at health, but only accounts for criminal activity, not other domains of risk despite the fact that FFT addresses multiple domains |
| Bratanova 2014 (Australia)[55] | 110 at risk young people; those likely to become clients of the justice system  12-17 yrs | Hypothetical implementation of Justice Reinvestment (JR) diversion program based on redirection of government funding to JR  Two options (1.conservative & 2.optimistic) based on AU$10 million (at 2013 prices) initial investment over 4 years. Impact realised by 2020, 5 years after inception. Numbers in corrective services & those receiving youth justice supervision will decrease & number of families provided with support will increase. | Criminal activity | Reduced crime | Hypothetical CEA  Comparators:  business as usual (BAU) vs justice re-investment  Perspective: QLD govt. | Present value of BAU 2015-2030 is AU$8.862 billion (at 2013 prices).  **JR Option 1:** Out of 110 youth, 7 should avoid offences leading to community -based supervision & 1 to detention each year. 6 people should avoid imprisonment & 15 should be removed from community corrections each year.  **JR Option 2:** Out of 200 youth who receive intensive family support, 10 would offend and be in detention based supervision & 20 in community-based supervision. 2 people would avoid imprisonment & 4 would not serve their sentence in community corrections.  Suggestion that if community services were only 5-10% efficient in the prevention of youth offences & at least 1-2% efficient in preventing young people from entering corrective services, it would save QLD government AU$263 million by 2030 (at 2013 prices; 3.5% discount rate) |
| Wang 2000 (US)[34] | 345 high school students from 10 schools (5 randomised to intervention; 5 to control) in northern California & the same for 10 schools in SE Texas  12-18 yrs | Safer Choices, a school-based HIV, other STDs & teen pregnancy prevention intervention for high school students | Sexual behaviour | Increase in condom use (STDs & pregnancies averted); | Retrospective CEA  Comparator: no intervention  Perspective: societal | At base case assumptions & an intervention cost of US$105,243 (at 1994 prices), Safer Choices achieved a 15% increase in condom use & an 11% increase in contraceptive use within one year. An estimated 0.12 cases of HIV, 24.37 cases of chlamydia, 2.77 cases of gonorrhoea 5.86 cases of pelvic inflammatory disease & 18.5 pregnancies were averted.  Data from 7 month follow up only; some cost data outdated or missing; assumptions about heterosexual sex only  No directly measured data re STDs and pregnancies averted so modelling used. Bernoulli model of HIV transmission (cumulative probability) used to translate condom/contraception use into HIV & other STDs averted. Pregnancy model also developed to translate condom/contraception use into pregnancies averted. |
| Sheidow 2004 (US)[50] | 154 youth  presenting with psychiatric emergencies in  South Carolina  10-17 yrs | RCT - randomly assigned to MST intervention or TAU (hospitalisation followed by usual aftercare community services) (Henggeler et al. 1999) | Mental health & wellbeing | Emotional distress (Global Severity Index of the Brief System Inventory); externalising & internalising behaviours (Child Behaviour Checklist) | Retrospective CEA  Comparator: TAU (hospitalisation + usual aftercare community services) Perspective: Program | 1 point improvement in externalising behaviours associated with a cost of US$395 (at 2000 prices) for MST recipients whereas for same improvement, TAU incurred a cost of $1527. Equivalent costs & outcomes after 12 months, therefore MST has better short term cost-effectiveness than TAU; but equivalent long-term cost-effectiveness |
| Lynch 2005 (US)[48] | 94 at-risk youths with depressive parents  Kaiser Permanente Northwest, Portland, Oregon  13-18 yrs | Preventive group CBT for adolescents with depressive parents  RCT conducted by Clark et al  CBT group + usual care (n=49) vs usual care for adults (n=45) | Mental health & wellbeing | Depression free days  QALYs | Retrospective CEA  Comparator: usual care for adults  Perspective Societal | ICER US$10 per depression free day (DFD) or US$9275 (at 2000 prices) per QALY (base case analysis). CEAC showed that at a willingness to pay of US$20 per day, the probability of the CBT intervention being cost effective was about 75%  Small group at one health centre. Not generalizable. CEA conducted during the12 months after intervention. No long term effect measured. |
