# Supplementary material for: Systematic review of economic evaluations of interventions for high risk young people
Source: BMC Health Serv Res. 2018 Aug 23;18:660. doi: 10.1186/s12913-018-3450-x (PMC6108123; doi:10.1186/s12913-018-3450-x)
Supplement: Supplementary file 3 — Quality assessment of economic evaluations using the Drummond checklist. (DOCX 18 kb) [file 12913_2018_3450_MOESM3_ESM.docx]

**Additional file 3: Quality assessment of economic evaluations using the Drummond checklist**

| **Study** | **Research question well defined?** | **Comprehensive description of alternatives?** | **Effectiveness of program established?** | **Important & relevant costs & consequences for each alternative identified?** | **Costs & consequences measured accurately & appropriately?** | **Costs & consequences valued credibly?** | **Costs & consequences adjusted for differential timing?** | **Incremental analysis of costs & consequences performed?** | **Allowance made for uncertainty in estimates?** | **Presentation & discussion of study results include all issues of concern to users?** | **Score** |
| --- | --- | --- | --- | --- | --- | --- | --- | --- | --- | --- | --- |
| Belfield 2003 | **✓** | **✓** | **✓** | **✓** | **✓** | **✓** | **🗶** | **🗶** | **🗶** | **✓** | **Average** |
| Rosenthal 2009 | **✓** | **✓** | **✓** | **✓** | **✓** | **✓** | **✓** | **🗶** | **✓** | **✓** | **Good** |
| Zerbe 2009 | **✓** | **✓** | **✓** | **✓** | **✓** | **✓** | **✓** | **🗶** | **✓** | **✓** | **Good** |
| SVA Consult. 2013 | **✓** | **✓** | **✓** | **✓** | **🗶** | **🗶** | **✓** | **🗶** | **✓** | **✓** | **Average** |
| Access Econ. 2008 | **✓** | **🗶** | **✓** | **✓** | **🗶** | **🗶** | **✓** | **✓** | **✓** | **🗶** | **Average** |
| Kuklinski 2012 | **✓** | **✓** | **✓** | **✓** | **✓** | **✓** | **✓** | **🗶** | **✓** | **✓** | **Good** |
| Hoeflmayr 2008 | **✓** | **✓** | **✓** | **✓** | **✓** | **✓** | **✓** | **🗶** | **✓** | **🗶** | **Good** |
| Guyll 2011 | **✓** | **🗶** | **✓** | **✓** | **✓** | **✓** | **✓** | **✓** | **✓** | **✓** | **Good** |
| French 2003 | **✓** | **✓** | **✓** | **✓** | **✓** | **✓** | **🗶** | **🗶** | **🗶** | **✓** | **Average** |
| Robertson 2001 | **✓** | **✓** | **✓** | **🗶** | **✓** | **🗶** | **🗶** | **✓** | **🗶** | **✓** | **Average** |
| Cartwright 2009 | **✓** | **✓** | **✓** | **✓** | **✓** | **🗶** | **🗶** | **🗶** | **🗶** | **✓** | **Average** |
| Klietz 2010 | **✓** | **✓** | **✓** | **✓** | **✓** | **🗶** | **🗶** | **🗶** | **🗶** | **✓** | **Average** |
| Wang 2000 | **✓** | **✓** | **✓** | **✓** | **✓** | **✓** | **✓** | **✓** | **✓** | **✓** | **Good** |
| Dealy 2013 | **✓** | **🗶** | **✓** | **✓** | **✓** | **✓** | **🗶** | **🗶** | **✓** | **✓** | **Average** |
| Sheidow 2012 | **✓** | **✓** | **✓** | **✓** | **✓** | **✓** | **🗶** | **🗶** | **🗶** | **✓** | **Average** |
| Wang 2001 | **✓** | **✓** | **✓** | **✓** | **✓** | **✓** | **✓** | **✓** | **✓** | **✓** | **Good** |
| Ross 2006 | **✓** | **✓** | **✓** | **✓** | **✓** | **✓** | **✓** | **🗶** | **✓** | **✓** | **Good** |
| Dino 2008 | **✓** | **✓** | **✓** | **✓** | **✓** | **🗶** | **✓** | **✓** | **✓** | **✓** | **Good** |
| Vijgen 2008 | **✓** | **✓** | **✓** | **✓** | **✓** | **🗶** | **✓** | **✓** | **✓** | **🗶** | **Good** |
| Jit 2009 | **🗶** | **🗶** | **🗶** | **✓** | **✓** | **🗶** | **✓** | **✓** | **✓** | **✓** | **Average** |
| Hollingworth 2012 | **✓** | **✓** | **✓** | **✓** | **✓** | **✓** | **🗶** | **✓** | **✓** | **✓** | **Good** |
| Swisher 2004 | **✓** | **🗶** | **✓** | **✓** | **✓** | **✓** | **🗶** | **🗶** | **✓** | **✓** | **Average** |
| French 2008 | **✓** | **🗶** | **✓** | **✓** | **✓** | **✓** | **🗶** | **🗶** | **🗶** | **✓** | **Average** |
| Dennis 2004 | **✓** | **✓** | **✓** | **✓** | **✓** | **✓** | **🗶** | **🗶** | **🗶** | **✓** | **Average** |
| Ingels 2013 | **✓** | **✓** | **✓** | **🗶** | **✓** | **✓** | **🗶** | **✓** | **🗶** | **✓** | **Average** |
| Schawo 2012 | **✓** | **🗶** | **🗶** | **✓** | **✓** | **✓** | **✓** | **✓** | **✓** | **✓** | **Good** |
| Bratanova 2014 | **✓** | **✓** | **✓** | **✓** | **✓** | **✓** | **✓** | **🗶** | **✓** | **✓** | **Good** |
| Sheidow 2004 | **✓** | **✓** | **✓** | **✓** | **✓** | **✓** | **🗶** | **🗶** | **🗶** | **✓** | **Average** |
| Lynch 2005 | **✓** | **✓** | **✓** | **✓** | **✓** | **✓** | **🗶** | **✓** | **✓** | **✓** | **Good** |
